# Translational Bioinformatics Applied to the Study of Complex Diseases

**DOI:** 10.3390/genes14020419

**Published:** 2023-02-06

**Authors:** Matheus Correia Casotti, Débora Dummer Meira, Lyvia Neves Rebello Alves, Barbara Gomes de Oliveira Bessa, Camilly Victória Campanharo, Creuza Rachel Vicente, Carla Carvalho Aguiar, Daniel de Almeida Duque, Débora Gonçalves Barbosa, Eldamária de Vargas Wolfgramm dos Santos, Fernanda Mariano Garcia, Flávia de Paula, Gabriel Mendonça Santana, Isabele Pagani Pavan, Luana Santos Louro, Raquel Furlani Rocon Braga, Raquel Silva dos Reis Trabach, Thomas Santos Louro, Elizeu Fagundes de Carvalho, Iúri Drumond Louro

**Affiliations:** 1 Departamento de Ciências Biológicas, Universidade Federal do Espírito Santo, Vitória 29075-010, Espírito Santo, Brazil; 2 Departamento de Medicina Social, Universidade Federal do Espírito Santo, Vitória 29040-090, Espírito Santo, Brazil; 3 Escola Superior de Ciências da Santa Casa de Misericórdia de Vitória (EMESCAM), Vitória 29027-502, Espírito Santo, Brazil; 4 Instituto de Biologia Roberto Alcantara Gomes (IBRAG), Universidade do Estado do Rio de Janeiro (UERJ), Rio de Janeiro 20551-030, Rio de Janeiro, Brazil

**Keywords:** Translational Bioinformatics (TBI), complex diseases, cancer, omics, big data

## Abstract

Translational Bioinformatics (TBI) is defined as the union of translational medicine and bioinformatics. It emerges as a major advance in science and technology by covering everything, from the most basic database discoveries, to the development of algorithms for molecular and cellular analysis, as well as their clinical applications. This technology makes it possible to access the knowledge of scientific evidence and apply it to clinical practice. This manuscript aims to highlight the role of TBI in the study of complex diseases, as well as its application to the understanding and treatment of cancer. An integrative literature review was carried out, obtaining articles through several websites, among them: PUBMED, Science Direct, NCBI-PMC, Scientific Electronic Library Online (SciELO), and Google Academic, published in English, Spanish, and Portuguese, indexed in the referred databases and answering the following guiding question: “How does TBI provide a scientific understanding of complex diseases?” An additional effort is aimed at the dissemination, inclusion, and perpetuation of TBI knowledge from the academic environment to society, helping the study, understanding, and elucidating of complex disease mechanics and their treatment.

## 1. Introduction

Computational biology, in recent decades, has gradually become more relevant in biological sciences [1], due to the significant decrease in cost of next-generation technologies and the progressive insertion of bioinformatics in medicine and translational research, playing a significant role in enhancing various biological studies [1,2].

These technologies have been adopted worldwide by numerous research groups and are being used to identify new Mendelian disease genes, while next-generation sequencing (NGS) is reaching routine clinical diagnostics [2]. However, translating the genome to the clinic depends on cross-referencing large amounts of data and various omics, as well as considering the environmental influence in these processes. Translational Bioinformatics’s (TBI) main objective is to translate results obtained from data processing into clinical practice. This requires the effort of a multidisciplinary team and the creation of computational models capable of enabling such a task [3].

Although its precise origin is uncertain, bioinformatics emerged from two main developments: (i) the increased understanding of the form and function of biological molecules, starting from results obtained from 1930s electrophoresis technique, and the consecutive discoveries involving DNA, RNA and protein structure; (ii) parallelly, the increase in computational power, such as mainframe applications (in the 1950s) and, later, modern workstations, as well as a continuous growth in the number of bioinformatics professionals, allowing for the understanding of numerous biological problems [4].

The identification of genes associated with human diseases is a determining factor for disease diagnosis in medicine [5]. In recent years several high-throughput techniques have been used to search for genes that cause common diseases, such as gene expression profiling, single nucleotide polymorphism (SNP) typing, whole genome sequencing, exome, total RNA sequencing (RNAseq), and protein sequencing [6]. However, there is a deadlock related to the use of such techniques, as they usually produce hundreds of candidate genes related to the disease being investigated [6]. To solve this problem, computational approaches, particularly network-based approaches, have been developed to efficiently determine disease-associated genes from existing biomedical networks [5], enabling data clustering and integration in order to build an accurate understand how cells work [4].

The creation of databases with biological information was the most important development to support the emergence of Bioinformatics. In the 1970s, structural biologists, using X-ray crystallography techniques, built the Protein Data Bank (PDB) specifying the Cartesian coordinates of the structures they elucidated, and made the PDB available to the public. As the ability to sequence DNA molecules became more affordable, DNA databases increased in number and quality. In the mid-1980s, the GenBank database was developed as a gene sequence information repository from the National Institutes of Health, an annotated collection of all publicly available DNA sequences [4].

Advances in research methods have resulted in experiments that generate vast amounts of data, which pose new challenges for researchers in storing, analyzing, and sharing data efficiently and appropriately [4]. In this context, the concept of Big Data arises, referring to a complex and large amount of data defined by: (i) large volume; (ii) diversity: stored data format can be unstructured and structured text, images, metadata, among others; (iii) speed of analytical processing; and (iv) data veracity or uncertainty: very large data collections can often combine several sources of varying reliability and trustworthiness [7]. In biological sciences, Big Data has been employed in enhancing curation, efficient analysis, and accurate interpretation, as well as accurate modeling and prediction of complex data [1].

In the age of Big Data, TBI can produce new methods and research knowledge about complex diseases, most notably cancer. To enable accurate and interpretable predictions, computational models based on protein structure analysis, methylation, expression, and activity of cancer hallmark signaling pathways are increasingly becoming the focus of current studies [8].

This review aims to guide, inform, and disseminate the understanding of TBI as a means to advance modern medicine. It highlights the benefits of understanding complex diseases, enabling training, specialization and abilities needed by various health professionals. We hope to answer the question: “How does TBI apply to and provide scientific understanding of complex diseases?”, and serve as a guide for those interested in this area.

## 2. TBI: From Definition to Application

TBI is a discipline that has come to the forefront of science in recent years because of the many beneficial results in solving and treating complex diseases. According to Tenenbaum [9], TBI has become an important discipline in the era of personalized medicine, broadening the insights and hypotheses about previously untestable foci of study.

TBI is considered a hybrid field of study incorporating basic and clinical research, also being called biomedical informatics. Other fields also bridge basic and clinical research, such as imaging informatics, clinical informatics and public health informatics. As stated by Sarkar [10], these fields supported the transfer and integration of knowledge in the main domains of translational medicine, ranging from molecules to populations.

According to the American Medical Informatics Association (AMIA), TBI has succeeded in developing storage, analytical, and interpretive methods capable of optimizing the transformation of increasingly voluminous biomedical and genomic data into predictive data for the development of preventive medicine [9].

TBI is crucial for moving basic biological discoveries from the research bench to the patient care setting (clinical research), using clinical information to understand basic biology. Likewise, TBI focuses on patient care, including the creation of new diagnoses, prognoses, prevention strategies, and therapies based on biological discoveries (Table 1) [11].

Systems biology is a modern and advanced field of study, focusing on understanding the behavior of entire biological systems, not just individual components. It uses methods ranging from qualitative network diagrams that link molecular and phenotypic entities, to formal quantitative models based on differential equations [11].

TBI research has started to develop Translational Biomedical knowledge in order to form a common language among researchers from different fields, connecting genomic information with phenotypic correlation analysis, enabling this increasingly transdisciplinary field [10].

In the last two decades, along with TBI and systems biology, the analysis and modeling of biomedical data with a network structure has emerged, supported by numerous network-based learning methods that have been developed in order to select one that learns and adapts to clinical application, resulting in highly meaningful, accessible, low-cost, and less time-consuming data analysis (Table 1) [12].

**Table 1:** TBI application in complex diseases.

Authors	Study Description
Ahmed [13]; Kang; Ko; Mersha [14]; Savoska; Ristevski; Trajkovik [15].	Integration of collective and individualized clinical data with patient-specific multi-omic data, AI algorithms and cloud electronic health record databases.
Baruah; Deka; Mahanta [16].	Multidisciplinary cooperation between laboratory and clinical researchers, aiming to meet community needs.
Bellazzi et al. [17].	Clinical bioinformatics (CBI) seeks to integrate molecular and clinical data, using bioinformatics to understand molecular mechanisms and potential therapies.
Bruggemann et al. [18].	Pharmacogenomics provides personalized patient care by selecting specific drugs for diseases, such as non-small cell lung cancer.
Han; Liu [19].	AI unravelling latent data behavior and generating new insights and optimal strategies in decision-making.
Liu et al. [20].	TBI used in reproductive medicine.
Liu; Wang; Lai [21].	Single-cell total RNA sequencing (scRNA-seq) and bulk total RNA sequencing (RNA-seq) associated with machine learning for detection of tumor origin.
Lussier; Butte; Hunter [22].	TBI effects on: (i) availability and cost reduction of molecular measurements; (ii) accessibility to measurements of health and disease status; (iii) sharing data and molecular tools; (iv) interpretation of new clinical molecular discoveries; (v) research funding.
Mitra et al. [23].	In silico analysis of acquired, archived, and retrieved biological data, as well as dynamic molecular docking experiments affecting pharmacology, biotechnology, bioengineering and education.
Sheikh; Ramlal; Khan [24].	Predictive modeling of antineoplastic drugs through computational analysis of microarrays.
Tang et al. [25].	TBI encompassing issues of equity and inclusion, by means of phenotyping applications, characterization of disease subtypes, predictive modeling, biomarker discovery and selection of better treatments.
Torkamannia; Omidi; Ferdousi [26].	Combined pharmacotherapy with synergistic/additive effect as a powerful treatment strategy for complex diseases.
Yang et al. [27].	High-throughput technologies/TBI in the biological interpretation of Big Data, uncovering mechanistic landscapes of complex diseases.

## 3. Translational Biomedical Knowledge

Translational Biomedical Knowledge and TBI have been shown to be highly efficient in developing new insights and proposing new hypotheses that were previously untestable. The 2019 Yearbook of the International Medical Informatics Association (IMIA) demonstrates TBI trends, on various aspects of bioinformatics methods and techniques to advance clinical care [28,29].

This translational knowledge has enabled work to be done at a low cost and in less time, because much of the recent discussion from bench to bedside research has been focused on how to allocate limited resources in support of science, in order to generate transformative clinical impact [30]. According to Shameer et al. [31], by characterizing health status through individual translational knowledge, earlier identification of true and personalized pathological changes can occur, avoiding unnecessary testing after accidental findings.

This knowledge can turn genomic data into clinically actionable knowledge, allowing for the understanding of complex drug interactions. Shameer et al. [31] explain that by integrating data from deep molecular profiling technologies (genomic, transcriptomic, proteomic or metabolomic), collectively defined as multi-omics data with clinical information, authors were able to explain some of the clinical variations between individuals and improve the use of health monitoring data for prediction, diagnosis, and development of intelligent clinical decision support systems, helping a more comprehensive understanding of clinical pharmacodynamics, pharmacokinetics, and the molecular mechanisms underlying drug effects, achieving relevant clinical utility and lower treatment side effects [32].

## 4. Precision Medicine and TBI

The objective of precision medicine is to use genetic data to improve health care decision-making [33,34]. Considering the growing volume of data and information generated, TBI can contribute considerably to the evolution of precision medicine. Since the primary goal of precision medicine is to find a dynamic treatment regimen that works well in future patients, Kosorok and Laber [34] highlight the usefulness of machine learning methods.

Recent studies have pointed to omics data importance and the potential in precision medicine. Xiao and colleagues [35] demonstrated how combined omics data from triple negative breast cancer (TNBC) has linked its metabolome and genomics. Authors pointed out the importance of TNBC metabolomic data for its precision treatment [35]. Milluzzo and collaborators [36], in a review paper addressing the clinical management of patients with diabetes and cancer, highlight the importance of a personalized, patient-centered, multidisciplinary and shared approach in the treatment of complex diseases, leading to the optimization of human and financial resources, and obtaining better clinical results [36].

Complex and voluminous datasets can be used through TBI to provide information that will enable better clinical outcomes. Qazi and Raza [37] point out that TBI has the potential to evolve traditional domains of biomedical sciences to improve health care infrastructure management and regulation [37].

## 5. Omics Revolution in Complex Diseases

The revolution in molecular biology caused the need to analyze previously unprecedented large amounts of data, requiring biology to make a transition from a more qualitative science to a true data science [38]. Organizing, integrating, and understanding have become the watchwords for multi-omics studies associated with understanding the biology of complex diseases. While the number of multi-omics studies has increased rapidly in recent years, the diversity of methods for integrating these data remains limited, being purely data driven. In order to advance in this field, there is a need to capitalize on the extensive prior knowledge about component interactions, and create a mechanistic understanding tool [39].

With the integration of omics approaches, the unbiased analysis of the whole genome (genomics), transcriptome (transcriptomics), proteome (proteomics), and metabolome (metabolomics) from different types of samples has been achieved [40]. The integration of the genome and transcriptome has offered an unprecedented opportunity to determine unambiguous genotype-phenotype correlation, such as the integration of genomic variation (e.g., single nucleotide polymorphism–SNP) and transcriptional alteration in single cells [41].

The integration of transcriptome with proteome has allowed phenotypes to be fully defined based on gene expression, cellular metabolism, structural dynamics, and signal transduction [41]. In the case of metabolomics integration, it was possible to perform targeted or unbiased identification of endogenous metabolites from body fluids, correlating metabolism, epigenomic changes, and proteomics [40]. There has been a transition from focusing on a small number of genes at a time, to simultaneously measuring as many of these cellular components as possible, significantly elevating the biological datasets collected simultaneously [38].

According to Subramanian et al. [42], the addressable biological questions on the vision of multi-omics data integration are broadly categorized into three different case studies: (i) disease subtyping and classification; (ii) predicting biomarkers for various applications, including diagnostics; (iii) gaining insights into disease biology.

The subtyping and classification of samples based on their omics profiles, especially cancer, are heterogeneous due to the remarkable differences in disease progression in affected individuals. Therefore, identifying the underlying subtypes of a disease or classifying samples into known subgroups makes it possible to understand disease etiology and identify appropriate interventions for patients belonging to different subgroups. Biomolecules are tightly interconnected, providing the flow of information of biological processes. Understanding the mechanistic details of disease biology is critical to the diagnosis and development of new therapeutic interventions [42].

**Figure 1:**
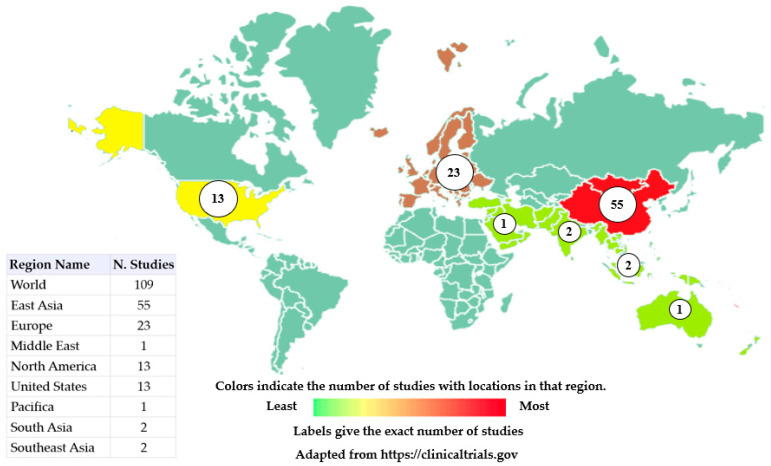
Global distribution of clinical trials using terms Omics and Cancer. Clinical studies that have omics and cancer as keywords are shown here. Studies in East Asia correspond to 50.46% of clinical trials, European studies correspond to 21.10%, and United States correspond to 11.92% of all clinical trials. Colors indicate the number of studies located in the region, ranging from green (least studies) to red (most studies). Studies with no location are not included in the counts or on the map and studies with multiple locations are included in all corresponding regions.

Among the complex diseases addressed in this manuscript, cancer stands out as the main purpose of our review. To achieve this goal, we have searched https://clinicaltrials.gov (accessed on 9 January 2023) and 109 studies were found for the terms “Omics” and “Cancer”. Figure 1 shows the distribution of clinical trials around the globe (Asia, Europe and the United States). The most relevant trials are shown in Table 2.

## 6. Integrating Complex Diseases through TBI

Ontologies are used to document new knowledge from biological and biomedical research, from classical biochemical experiments to omics experiments. These ontologies are created, maintained, and extended by experts, with the goal of providing a unified annotation schema that is human- and machine-readable [43]. According to The Gene Ontology Consortium [44], the ontology encompasses three divisions, these being: (i) molecular function (the activity of a gene product at the molecular level), (ii) cellular component (the location of a gene product’s activity in relation to biological structures), and (iii) biological process (a larger biological program in which the molecular function of a gene is used). According to these divisions, ontology enables complex answers to be obtained in a simplified way, being treatable by both man and machine, in order to offer reliable data according to the system addressed.

Manda [45] addressed the use of association rule mining focused on predicting annotations, becoming crucial to discover new relationships between ontologies and other applications. Wang [46] and Quan [47] demonstrated the use of ontology data in pathway analysis (organizing and eliminating pathway data redundancy), which are crucial for understanding physiology and pathogenesis of diseases. Schriml et al. [48] applied a human disease ontology (DO) to provide disease classification by formal semantic rules to specifically express meaningful disease models, aiming to include mechanistically inferred multiple-disease classifications, enabling new insights into related diseases, such as the heterogeneity of genetic diseases and the multicellular origin of cancer.

## 7. Application of TBI in Complex Diseases

Applications of TBI are evidenced by Roy, Singh and Gupta [49], who highlighted an innovative study against pancreatic ductal adenocarcinoma, through an integrated analysis of DNA methylation and gene expression datasets aiming at better mechanistic and molecular insights that can be correlated with clinical data. These authors provide valuable results for prognostic improvements, personalized treatment and delineation of the heterogeneous landscape of pancreatic ductal adenocarcinoma, and may enable personalized therapies and risk prediction.

According to Liu et al. [50], a new connection of TBI with validation through in vitro, in vivo, and patient-derived samples has proven possible in the face of breast cancer biomarker discovery. They used circulating non-coding RNA as a source of new biomarkers for non-invasive screening. As a consequence, hsa-miR-423-5p expression in plasma and blood exosomes of breast cancer patients was observed to be abnormally high compared to healthy controls. Encoding genes regulated by hsa-miR-423-5p were widely distributed in signaling pathways associated with tumors in silico.

Cai et al. [51] incorporated improvements in analytical methods for the detection of differentially expressed genes (DEGs) between two different phenotypes with limited sample sizes, enabling improvements in reaching vital clues for cancer treatment. Chen et al. have developed a highly effective tool to accurately detect and visualize gene fusions, which play an important role in cancer.

Yu, Zhao and Gao [52] used miRNA data on target genes and disease tissue specificity, as well as information from the Food and Drug Administration, to construct drug-miRNA-disease networks, and potential disease treatment prediction, which was employed in breast cancer cases, detecting new potential drugs for treatment. Zeng et al. [53] built a co-expression network using a gastric cancer model, and performed enrichment analyses to identify key unique genes, suggesting them as likely biomarkers of cell subtype. Likewise, Zhou et al. [54] demonstrated green tea data on suppression of proliferation pathways in cancer, as well as positive regulation for certain miRNAs, in a study combing miRNA, mRNA, pathway and network analysis.

TBI has highlighted several new and improved studies for other complex diseases, such as rare diseases, metabolic syndrome, and pandemic diseases, and has served as a pillar for new research on biological networks, such as co-expression networks and multi-omics analyses. In this context, Akgün et al. [55] addressed the importance of TBI for rare disease analyses and preservation of genomic data for these patients, due to the difficulty of obtaining biological data.

Immel et al. [56] portrayed the use of genomic DNA analysis from buried victims who had been afflicted by the medieval plague. Given its devastating effect, the second plague pandemic caused by *Yersinia. pestis* was a strong candidate to exert selective pressure on the human immune response. Thus, the authors developed a study that indicated that the differences in allele frequencies of HLA genes involved in innate and adaptive immunity (responsible for extracellular and intracellular responses) to pathogenic bacteria (such as *Yersinia. pestis*) may have been affected by the historical epidemics that occurred in Europe in the past.

Recently, humanity experienced a major pandemic caused by the SARS-CoV-2 virus, which, due to the lack of available drugs or vaccines, made the rapid virus spread and progress throughout the world, causing many deaths. TBI proved to be useful and innovative by performing a crosstalk between molecular modeling techniques, molecular docking, and in vitro testing, as described by Pooja et al. [57], who made the ability of in silico studies to provide candidate molecules for antiviral drug development explicit.

Jaballah et al. [58] coupled in silico studies via biological networks with molecular analyses for understanding menopausal hormonal changes associated with the onset of metabolic syndrome (MS) and its consequences for type 2 diabetes (T2DM) and cardiovascular disease (CVD). To achieve this goal, the authors exploited a TBI approach to detect common genetic signatures for MS, DM2 and CVD, and menopausal status, and, through enrichment analysis, provided core genes that may play a key role in menopausal status and influence the risks of MS, DM2, and CVD.

Based on Djeddi et al. [59], TBI has also been used in multi-omics approaches finding different molecular signatures common to diverse diseases and therapeutic strategies. Huang et al. [60] highlighted the high demand of using gene co-expression network (GCN) mining, in such way that TBI identified gene modules with correlated expression profiles. These interactions have made it possible to discover new latent genetic interactions, and new gene functions, and to extract molecular features of certain disease groups, finding new disease biomarkers.

Thalor et al. [61] highlighted the impact of a translational approach on the elucidation of potential gene signatures, such as genes associated with MAPK, PI3-AkT, Wnt, TGF-β and other signal transduction pathways, to demonstrate new molecular hypotheses about the metastasis process related to triple negative breast cancer (TNBC). Ullah et al. [62] highlighted the ability of bioinformatics to provide better diagnostic and therapeutic markers for colorectal cancer, being able to identify the effectiveness of SRY-Box Transcription Factor 9 *(SOX9*) in improving colorectal cancer prognosis.

Kaur [63] applied a TBI approach to the study of gliomas to obtain better molecular classification of different grades of glioma and demonstrated the need for in-depth assessments on critical genes for cancer development. Yi et al. [64] applied TBI on mantle cell lymphoma by including sequencing of patient longitudinal samples and RNA-seq data, obtaining genetic subsets that could guide a clinical understanding of cancer clonal evolution.

Yu et al. [65] have highlighted how connecting Bioinformatics with the clinic allows for the construction of a novel framework to evaluate and select assays to monitor cancer. They used NGS with large gene panels of somatic cancer mutations of circulating tumor DNA (ctDNA). In parallel, Xu et al. [66] pointed to the application of this area on the current COVID-19 pandemic research, in which TBI have helped understand potential disease mechanisms, and effective and less toxic treatments.

**Figure 2:**
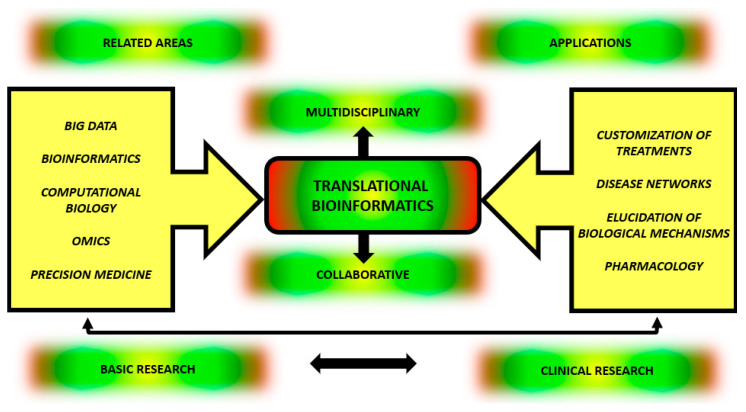
Integration between origin and outcomes of Translational bioinformatics. TBI applied to the study of complex diseases involves basic research and clinical research in a reciprocal, collaborative and multidisciplinary way. For correct understanding and treating complex multifactorial diseases, there is a need to integrate information from multiple areas, such as Big Data, bioinformatics, computational biology, molecular biology associated with omics data and precision medicine. By doing so, this knowledge can be applied to treatment customization, understanding disease networks, elucidating biological processes and developing translational pharmacology.

Last, but not least, Battineni et al. [67] emphasized an improvement on sample storage for future research through the use of TBI, because this area provides a crucial means of applying artificial intelligence on data analysis, disease diagnosis, prediction and classification of pathological findings. These numerous applications underscore the unequivocal innovative role of TBI in various areas of medicine in an interdisciplinary and multidisciplinary way, as symbolized in Figure 2.

### 7.1. Relationship between Next-Generation Sequencing (NGS) and TBI in the Study of Complex Diseases

The constant evolution of sequencing technologies has motivated modern bioinformatics, shaping the area of basic and clinical research with advanced techniques based on sophisticated computation, artificial intelligence, machine, and deep learning [68].

The development and application of next-generation sequencing platforms enabled bioinformatics improvements primarily through sequencing automation methods, mainly due to the needs of personal genomes and metagenomics projects [68].

Sequencing is subdivided into generations: (i) first generation (Sanger sequencing), provides high precision and helps in validating NGS discoveries, but has a low yield; (ii) second generation, with high throughput, short reading length, low cost, difficult sample preparation, clinical applications and PCR amplification; (iii) third generation, highlights the absence of PCR amplification, requires less initial material, longer reading lengths, very low cost and low error rate during library preparation, but permeates a relatively high sequencing error rate and a small number of algorithms/tools for final analysis; and (iv) fourth generation, ultra-fast scanning of the entire genome, enabling sample spatial distribution reads (in situ sequencing) [69].

Among numerous applications of this crosstalk between NGS and bioinformatics, clinical genetics has achieved numerous improvements regarding the analysis of hundreds of genes at an unprecedented speed and low cost, applying bioinformatics algorithms to deal with complex and heterogeneous disorders by combining information from multiple omics sources (such as genome, transcriptome, proteome and epigenome), to develop new machine learning algorithms, aiming at improving NGS utility and performance, achieving superior clinical diagnostics and opening new therapeutic paths [70].

In addition, third-generation sequencing has demonstrated innovative translational solutions for the diagnosis of infectious diseases (pathogen detection and characterization of mixed microbial communities) [71], cancers and other diseases, identifying a large number of disease variants in the human genome [72], using a relatively low-cost platform, fast response time and easy-to-use bioinformatics pipelines [71], thus, generating an increase in disease molecular diagnostic accuracy, using unique and real-time molecular sequencing technologies [73].

Exome sequencing has been providing an increase in disease diagnostic yield, identifying new pathogenic genetic variants [74].

Bioinformatics coupled with high computational power through cloud platforms, offers scalability, safety and performance [75], integrating diagnostic and therapeutic tools for genomic and pharmacogenomic discoveries, in order to provide routine medical care, design specific drugs and personalized genome tests [76].

### 7.2. Multi-Omics, Single Cells, and TBI in the Study of Complex Diseases

The advances provided by sequencing associated with precision medicine have driven new paradigm shifts in clinical practice and basic research. The union of collective and individualized clinical data with patient-specific multi-omics data has resulted in new therapeutic strategies [13]. Multi-omics approaches allow for the integration of data from various platforms, in a multifaceted view of disease processes [77].

Single-cell omics provides a basis for data-driven reconstruction of cell lineage hierarchies, deepening the understanding of the underlying mechanisms that govern health and disease [77,78]. Bioinformatics plays a crucial role in the interpretation and analysis of single-cell data results. To achieve this, five complementary strategies are applied: (i) combine; (ii) separate; (iii) split; (iv) convert; and (v) predict [78].

While new research methodologies have been developed, new computational resources, along with new algorithms, mathematical models and new tools, have been used in multi-omics single-cell studies to uncover new information about complex diseases, such as cancer [77,78,79]. Nam, Chaligne and Landau [80] highlighted the multidimensional incorporation of omics with single cells in cancer, promoting better understanding of tumor evolution, unveiling the cell-to-cell genetic diversity, epigenetic profiles, spatial distributions, and microenvironment interactions.

## 8. Conclusions

TBI has the mission to investigate tumor biology from different perspectives, using novel approaches, combining cellular and molecular biology techniques, bioinformatics, and clinical data. By these means, it is possible to better understand and characterize tumor evolution, as well as develop new strategies for disease detection, control, and treatment. Research in this area aims to evaluate a large amount of data and understand molecular alterations in the cell genome that lead to the tumor phenotype, and evaluate the effects of these alterations on cell signaling and metabolic pathways, as well as the interaction of tumor biology with the immune system and its microenvironment. In the face of new discoveries in TBI, biomarkers for clinical and therapeutic use can be identified.

TBI research is characterized by being multidisciplinary, and it brings together expertise from different areas such as cell biology, molecular biology, genetics, immunology, virology, biochemistry, bioinformatics, and medicine, applying it in the pharmaceutical and biotechnology industry, in oncology translational research, and in the development of mathematical and computational models that can bring forward better treatment and quality of life for cancer patients.

Given the information presented, it appears that a joint effort is needed to increase the dissemination of knowledge about TBI from academia to society, highlighting the great benefit and impact of this area on the study, understanding, and elucidation of mechanisms for the treatment of complex diseases, especially cancer. In addition, it is important that higher education institutions offer students integrated and interdisciplinary training, bringing together the areas of computing, mathematics, statistics, biology, medicine, and other areas of health, aiming to train professionals to be able to act and develop research projects in TBI integrating basic and clinical research through diverse and dynamic content.

We hope to have provided an efficient guide for the dissemination of academic knowledge in this area for numerous audiences, especially students and health professionals.

## Figures and Tables

**Table 2:** Most relevant clinical trials using the terms Omics” and “Cancer”.

Study Title	Study Purpose	Conditions	Interventions	Locations	Study Number
Preliminary Study on Plasma Markers for Early Diagnosis of Lung Cancer	Molecular features of liquid biopsy and clinical data from lung cancer patients using multi-omics assays, validated a Machine Learning method able to discriminate lung cancer patients from healthy subjects.	Lung Cancer	Diagnostic test: A machine-learning tool to detect early-stage lung cancer.	China	NCT04558255
Advanced Therapies for Liver Metastases	State-of-the-art omics used to characterize the immune and non-immune microenvironment of primary tumor and liver metastases, correlating with activation status of peripheral blood leukocytes.	Pancreatic Ductal Adenocarcinoma, Colorectal Cancer, Liver Metastasis	Not provided.	Italy	NCT04622423
AI used to optimize early-stage hepatocellular carcinoma treatment based on multi-modal imaging.	AI omics imaging, traditional omics imaging and clinical information used to predict prognosis of different treatment options for early liver cancer.	Hepatocellular Carcinoma	Method: Contrast-enhanced ultrasound (CEUS) and Contrast-enhanced MRI examination (CEMRI).	China	NCT05627297
Electronic cigarettes cancer risk	Integrative multiomics approach used to determine the carcinogenic potential of e-cig, relative to cigarette smoking in oral epithelium.	Cigarette Smoking	Device: NIDA Standard Research E-cigarette (SREC)	United States	NCT03750825
Artificial Intelligence system for assessment of tumor risk, diagnosis, and treatment	AI used to establish a medical database of standardized and structured clinical diagnosis based on multiomics information.	Lung, Stomach, and Colon Cancer	Not provided	China	NCT05426135
Liquid Biopsy early detection of Gastric cancer	Prospective, observational, multiomics study focused on detection of gastric cancer by combined assays using serum protein markers, cfDNA deep sequencing, ctDNA mutation and circulating RNA. Bioinformatics tools like PEAR and Bismark are used to process FASTQ files.	Gastric Cancer	Diagnostic test: blood-based biomarkers	China	NCT05224596
Liquid biopsy early detection of pancreatic cancer (ASCEND-PANCREATIC)	Multiomic prospective study aims to detect early pancreatic cancer using combined assays for cfDNA methylation biomarkers, circulating tumor DNA (ctDNA) mutations, serum protein and miRNA markers using bioinformatics tools PEAR and Bismark.	Pancreatic Cancer	Not provided	China	NCT05556603
Breast Cancer, Omics, and Precision Medicine (BR(E)2ASTOME)	To evaluate the clinical utility of the early use of network-oriented BR(E)2ASTOME algorithm which combines the power of liquid-based assays, advanced epi-genomics, and network analysis to improve personalized therapies in BC.	Breast Cancer	Biological: Next-generation sequencing and network analysis	Italy	NCT04996836
Comprehensive Omics Analysis of Pediatric Solid Tumors/Repository for Related Biological Studies	To create a repository of blood, serum, tissue, urine, and tumors to perform omics profiling.	Sarcoma, Endocrine Tumors, Neuroblastoma, Retinoblastoma, Renal Cancer	Not provided	United States	NCT01109394
Concurrent Radiochemotherapy and Anlotinib for Locally Advanced Cervical Cancer	To observe the efficacy and safety of a new treatment option for cervical cancer. Multi-omics technology and bioinformatics tools were used to analyze the patients.	Uterine Cervical Cancer	Drug: Hydrochloride anlotinib, Drug: cis Platinum/carboplatin, Radiation: External beam radiotherapy and brachytherapy	China	NCT04772001
Lethal Prostate Cancer Biology–Urine Metabolomics	A prospective, observational and investigational study that aims to find new markers/targets for screening prostate cancer.	Prostate Cancer	Dietary supplement: Multi-carotenoids	Taiwan	NCT03237702
Artificial Intelligence System for Pathological Diagnosis and Therapeutic Effect Prediction Based on Multimodal Data Fusion of Common Tumors and Major Infectious Diseases in the Respiratory System.	To create a large medical database that includes standardized and structured clinical diagnosis and treatment information, and to develop a multi-modal data fusion-based technology system for individualized intelligent pathological diagnosis and therapeutic effect prediction using artificial intelligence technology.	Lung Cancer, Pulmonary Tuberculosis, Covid19	Not provided	China	NCT05046366
Early Diagnosis of Small Pulmonary Nodules by Multi-omics	To analyze the immunological repertoire and genetic mutations of pulmonary nodules using imaging tests, three-dimensional reconstruction, bioinformatics R-scripts and algorithms (OptiType algorithm).	Non-small-cell Lung Cancer	Not provided	China	NCT03320044
AI early screening of Colorectal Cancer Based on Plasma Multi-omics.	AI algorithm to evaluate accuracy and effectiveness of a novel screening method based on plasma multi-omics to detect colorectal cancer and advanced adenomas.	Colorectal Adenoma, Colorectal Cancer	Diagnostic test: Colonoscopy, Diagnostic test: ctDNA methylation, Diagnostic test: characteristics of ctDNA fragment	China	NCT05587452
Evaluation of Clinical Treatment of Multiple Myeloma Based on Multi-omics	Multi-omics method to find biomarkers of clinical efficacy, adverse reactions, and blood concentration of bortezomib in peripheral blood samples.	Multiple Myeloma, Bortezomib	Drug: Bortezomib	China	NCT04678089
Gut Microbiota and Metabolomic (MBS)	To evaluate the correlation between intestinal microbiota and metabolites in Borrmann type IV gastric cancer and to use Machine Learning to build models of intestinal microbiota and metabolomics.	Stomach Neoplasms, Gut Microbiota, Metabolomics	Procedure: Healthy control specimen collection, Procedure: Non-Borrmann IV patient specimen collection, Procedure: Borrmann IV patient specimen collection	China	NCT05205187
I3LUNG: Integrative Science, Intelligent Data Platform for Individualized lung cancer immunotherapy	AI international project that aims to predict immunotherapy efficacy for NSCLC patients using the integration of multisource data (real-word and multi-omics data).	Lung Cancer	Not provided	United States, Greece, Israel, Spain	NCT05537922
Integrative Omics Analysis for Colorectal Cancer and Metastasis	Integrative omics to analyze and predict candidate biomarkers of colorectal cancer and distant metastasis.	Colorectal Cancer	Other: Integrative omics	China	NCT05482529
Multi-omics Characterization of Pancreatic Neuroendocrine Tumors	Integrated multi-omics to identify tumor subgroups in pancreatic neuroendocrine tumors and carcinomas regardless of their grade and stage.	Cancer of Pancreas	Not provided	France	NCT05234450
Multi-omics Sequencing in Neoadjuvant Immunotherapy of Gastrointestinal Tumors	To find new biomarkers of efficacy of combined immunotherapy.	Immunotherapy, Gastric Cancer, Rectal Cancer, Chemotherapy Effect, Radiotherapy	Drug: Terelizumab, Drug: CapeOx, Drug: Trastuzumab, Radiation: Radiotherapy	China	NCT05515796
Multi-Omics Noninvasive Inspection of Tumor Risk for Gastric Cancer	A prospective, case-control study intended to develop and validate a blood-based multi-omics assay and computational model for early detection of gastric cancer.	Gastric Cancer	Device: ctDNA multi-omics test	China	NCT04947995
Omics of Cancer: Onco Genomics	To create a registry of genomic/clinical data of cancer or cancer predisposition patients.	Neoplasms Cancer	Not provided	United States	NCT05431439
Pleural Carcinomatosis Tissue Banking	To create a biocollection of tissues from pleural carcinomatosis and characterize intratumoral heterogeneity through multi-omics and bioinformatics analysis.	Pleural Effusion, Malignant	Pleural biopsies	France	NCT04844827
Predictive Biomarkers in Patients with Advanced Hepatocellular Carcinoma Treated with Systemic Therapy	Multi-omics to find biomarkers of treatment response in hepatocellular carcinoma (HCC).	Hepatocellular Carcinoma	Drug: atezolizumab plus bevacizumab	Korea	NCT05197504
Project CADENCE (CAncer Detected Early caN be CurEd) (CADENCE)	To develop and validate multi-cancer screening tests based on multi-omics (single-cell early cancer detection algorithms).	Thoracic, Ovarian, Liver, Prostate, Gastric, Colorectal, Breast, Esophageal, and Pancreatic Cancer	Not provided	Singapore	NCT05633342
Prospectively Predict Gastrointestinal Tumor Treatment Efficacy Based on Peripheral Multi-omics Liquid Biopsy	To predict and monitor immunotherapeutic outcomes of gastrointestinal tumors.	Advanced Gastric Adenocarcinoma, Immunotherapy	Device: EV-array	China	NCT04993378
ML radiomic and pathomic study of Pituitary Adenomas	Machine learning to study multi-dimensional and multi-omics data, to train a risk prediction algorithm for refractory pituitary adenomas.	Pituitary Neoplasms	Diagnostic test: Artificial intelligence model	China	NCT05108064
Multi-omics immune prevention and treatment of gliomas	Omics sequencing and molecular biology technologies to study glioma treatment efficacy.	Transcriptomics, Radiomics, Glioma	Procedure: surgery	China	NCT04792437
Esophageal Cancer Neoadjuvant Chemoradiation response prediction using Artificial Intelligence & Machine Learning (QARC)	To predict treatment response in esophageal cancer patients using radiomics AI modeling.	Esophageal Cancer	Radiation: Neo-adjuvant radiotherapy, Drug: Neo-adjuvant chemotherapy, Procedure: Esophagectomy	India	NCT04489368
Easy-to-use Adrenal Cancer/Tumor Identity Card	To provide an easy-to-use “identity card” of adrenal tumors for personalized patient management.	Adrenal Gland Neoplasms	Biological: omics identity card	France	NCT02672020

## Data Availability

Not applicable.
